# Snapshot of a Bacterial Microbiome Shift during the Early Symptoms of a Massive Sponge Die-Off in the Western Mediterranean

**DOI:** 10.3389/fmicb.2016.00752

**Published:** 2016-05-19

**Authors:** Andrea Blanquer, Maria J. Uriz, Emma Cebrian, Pierre E. Galand

**Affiliations:** ^1^Centre d’Estudis Avançats de Blanes, Consejo Superior de Investigaciones CientíficasGirona, Spain; ^2^Departament de Ciències Ambientals, Facultat de Ciències, Universitat de GironaGirona, Spain; ^3^Laboratoire d’Ecogéochimie des Environnements Benthiques, Observatoire Océanologique de Banyuls, Sorbonne Universités, Université Pierre et Marie Curie, Université Paris 06, Centre National de la Recherche ScientifiqueParis, France

**Keywords:** mass die-off, keratose sponges, early disease symptoms, microbiome profiling, microbiome shifts, bacterial symbionts, *Ircinia fasciculata*, western Mediterranean

## Abstract

Ocean warming is affecting marine benthic ecosystems through mass mortality events that involve marine invertebrates, in particular bivalves, corals, and sponges. Among these events, extensive die-offs of *Ircinia fasciculata* sponges have been recurrently reported in western Mediterranean. The goal of our study was to test whether the temperature-related mass sponge die-offs were associated with or preceded by an early unbalanced bacterial microbiome in the sponge tissues. We took advantage of the early detection of disease and compared the microbiomes of healthy vs. early diseased *I. fasciculata* tissues. Our results showed a microbiome shift in early diseased tissues. The abundance of *Gammaproteobacteria* and *Acidobacteria* increased and that of *Deltaproteobacteria* decreased in diseased vs. healthy tissues. The change in community composition was also noticeable at the operational taxonomic unit (OTU) level. Diseased tissues contained more bacterial sequences previously identified in injured or stressed sponges and corals than healthy tissues. Bacterial diversity increased significantly in diseased tissues, which contained a higher number of low abundance OTUs. Our results do not support the hypothesis of one particular pathogen, whether a *Vibrio* or any other bacteria, triggering the Northwestern Mediterranean mass mortalities of *I. fasciculata*. Our data rather suggest an early disruption of the bacterial microbiome balance in healthy sponges through a shift in OTU abundances, and the purported consequent decline of the sponge fitness and resistance to infections. Opportunistic bacteria could colonize the sponge tissues, taking benefit of the sponge weakness, before one or more virulent pathogens might proliferate ending in the mass sponge die-off.

## Introduction

Ocean warming is affecting marine benthic ecosystems through epidemiologic diseases and mass mortalities of invertebrates, in particular bivalves, corals and sponges (Harvell et al., 2002). The mass mortality events are increasing across all oceans (Webster, 2007) and may lead to declines, or rises of other benthic species, with unpredictable subsequent effects (Pinnegar et al., 2000). Moreover, mass mortalities not only alter benthic assemblages but also may impair species harvesting and sea cultures with biotechnological purposes (Schippers et al., 2012).

The etiological agents responsible for marine mass mortalities have rarely been unambiguously identified (e.g., Angermeier et al., 2011, 2012). In corals, where diseases have long been studied (e.g., Rosenberg and Ben-Haim, 2002; Sutherland et al., 2004), the growing theory is that diseases are caused by opportunistic polymicrobial infections, rather than by a single primary pathogen (Lesser et al., 2007; Bourne et al., 2008; Rosenberg et al., 2009). Diseases usually follow an environmental perturbation (e.g., anomalous high temperatures) that produces physiological stress to the holobiont, which may unbalance the associated microbial community. Indeed, it has been proposed that coral diseases are caused by a change of some benign components of the associated microbial communities into pathogens (Lesser et al., 2007).

In sponges, several causative agents of the diseases have been proposed (Webster et al., 2002; Cervino et al., 2006; Webster, 2007; Luter et al., 2010; Stabili et al., 2012) but seldom unambiguously confirmed (Choudhury et al., 2015). Microorganisms involved in sponge diseases may be: (i) symbionts that turn into pathogens, often by increasing their abundance (i. e., Rützler, 1988; Olson et al., 2006, 2014); (ii) symbionts that change their virulence (but not necessarily their abundance) because of environmental changes (i.e., Smith, 1941); (iii) external microorganisms that interact with and modify the sponge symbiotic community (i.e., Webster et al., 2008); (iv) external common pathogens; (v) several combinations of the above-mentioned factors. Moreover, environmental factors can simply modify the composition of the symbiotic community (Lemoine et al., 2007; López-Legentil et al., 2008, 2010; Webster et al., 2008; Gao et al., 2015), which may reduce the host fitness and thus its resistance to external pathogenic microbes (Saby et al., 2009).

In the Mediterranean, mass mortality events, positively correlated with anomalously high seawater temperatures, have affected benthic filter-feeders and suspension-feeders recurrently (e.g., Cerrano et al., 2000; Perez et al., 2000; Coma et al., 2006; Garrabou et al., 2009). The most disturbed organisms have been gorgonians and sponges with proteinaceous skeletons (Perez et al., 2000; Garrabou et al., 2009), in particular representatives of Family Irciniidae (Maldonado et al., 2010; Cebrian et al., 2011; Stabili et al., 2012). *Ircinia fasciculata* (Pallas, 1766) is one of the most abundant Ircinidae in the shallow sublittoral, where it can reach large sizes and provide shading refuge for many benthic invertebrates including polychaetes, crustaceans, sipunculids, and small fishes (Koukouras et al., 1985, 1992). *I. fasciculata* belongs to the so-called bacteriosponges (i.e., High Microbial Abundance—HMA-sponges) (Blanquer et al., 2013), and harbors both phototrophic and heterotrophic bacteria. The former are mainly composed of one Cyanobacteria species densely distributed at the sponge periphery (Turon et al., 2013).

Extensive die-offs of *Ircinia* populations were reported during the 2008, 2009, and 2010 summers in the western Mediterranean (Maldonado et al., 2010; Cebrian et al., 2011), south Adriatic and Ionian Seas (Stabili et al., 2012), and North Adriatic (Di Camillo et al., 2013). The 2009 mortality represented one of the most severe sponge disease reported up to now as it affected up to 95% of the individuals of *I. fasciculata* from several marine protected areas of the western Mediterranean, with up to 100% of the individuals injured (Cebrian et al., 2011).

There are two main running hypotheses on the disease origin in Mediterranean *Ircinia* species based on indirect evidence. Some authors suggest that a twisted rod bacterium was involved in the disease, because of its presence in ultrastructure sections of diseased sponges (Maldonado et al., 2010). Other authors consider the bacterium *Vibrio rotiferianus* as a primary disease agent because it represented a higher proportion of bacteria in cultures from diseased individuals compared to healthy specimens (Stabili et al., 2012). However, none of these studies could refute that the putative etiological agents were not just opportunistic microbes that proliferated in stressed, damaged or dead sponge tissues (Gaino and Pronzato, 1989; Vacelet et al., 1994). Other authors tested experimentally the effect of higher temperatures on the physiology of the symbiotic cyanobacteria of *I. fasciculata*, and found that the sea temperatures recorded during mortality events strongly altered the cyanobacteria physiology (Cebrian et al., 2011). This study pointed to Cyanobacteria community shifts as a purported factor prompting the disease, since physiological alterations of Cyanobacteria occurred before the first external symptoms were detected (Cebrian et al., 2011).

Recent studies have emphasized the stability of the I. fasciculata associated bacteria through spatial and temporal scales (Erwin et al., 2012b; Pita et al., 2013a,b), and at high temperatures, but below the threshold producing sponge mortalities (Pita et al., 2013a). The sponge microbiome stability under sublethal thermal stress has also been reported for *Rhopaloeides odorabile* (Simister et al., 2012). Microbiome stability under changing conditions highlights the strength of these multiple symbioses and suggests that changes in the sponge microbiome may be a good proxy of sponge fitness decay and an indicator of a possible forthcoming sponge disease.

The main objective of this study was to test the hypothesis that sponge disease may be initiated by an unbalance in the sponge bacterial communities. We took advantage of the early detection of disease and compared the microbiome profiling of healthy and early diseased *I. fasciculata* tissues before opportunistic pathogens could have arrived. This is the first time that microbiomes from a mass mortality event of the sponge *I. fasciculata* have been captured before the disease symptoms were widely manifested.

## Materials and Methods

### Sampling

Three *I. fasciculata* sponges showing signs of disease were sampled during the 2010 summer disease episode from the Scandola Marine Protected Area (Corsica, France). All the sampled sponges later died. For each sponge, samples were collected from healthy tissues and from the area in close contact with small white spots. Sponge samples were put in individual tanks underwater and kept in the receptacles until fixed in absolute ethanol once on surface. Three replicates of 1.5 l seawater samples were also sampled from the vicinity of the sponges and filtered through three distinct 0.2 μm sterile filers (GE Osmonics, Minnetonka, MN, USA). The filters were maintained frozen at -80°C until DNA extraction.

**FIGURE 1:**
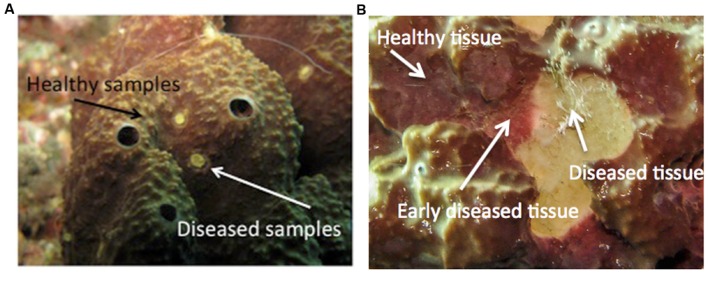
**Details of *Ircinia fasciculata* sponges at different stages of the disease.** **(A)** Early diseased individual (arrows indicate the tissue sampled). **(B)** An advanced disease stage (arrows point to healthy, what we consider early diseased, and advanced diseased tissues).

Healthy *I. fasciculata* have a dense external layer of the Cyanobacteria *Candidatus Synechococcus spongiarum* (Erwin et al., 2012c), which confers a characteristic yellowish to violet color to the sponge surface (**Figure 1**). We defined early diseased tissues the areas close to diseased tissues that show a lighter violet color. Conspicuously diseased tissues lacked the Cyanobacteria layer as confirmed by their whitish color but maintained their ectosome intact (**Figure 1**). To avoid a single Cyanobacteria operational taxonomic unit (OTU) flooding our bacterial dataset, the external layer of the sponge samples was removed before DNA extraction.

### DNA Extraction, PCR, and Sequencing

DNA from sponges was extracted with DNeasy blood and tissue kit (Qiagen; Blanquer et al., 2013). DNA concentration and quality were assessed using Qubit fluorometer (Invitrogen) and a Biomate spectrophotometer (Thermo Scientific), respectively. Extractions were performed on the inner sponge tissue to avoid the external Cyanobacteria-rich layer. As mentioned above, the aim was to prevent an over-representation of Cyanobacteria species, which would have decreased considerably the coverage of the heterotrophic bacterial communities. Bacterial 16S rRNA genes were amplified using primers 28F TTTGATCNTGGCTCAG and 519R GTNTTACNGCGGCKGCTG with a single-step 28 cycles PCR using the Hot Star Taq Plus Master Mix Kit (Qiagen). The PCR conditions were: 94°C for 3 min, followed by 28 cycles of 94°C for 30 s, 53°C for 40 s, and 72°C for 1 min, after which a final elongation step at 72°C for 5 min was performed. Following PCR, all amplicon products from the different samples were mixed in equal concentrations and purified using Agencourt Ampure beads (Agencourt Bioscience Corporation). Pyrosequencing was done with a Roche 454 FLX using commercially prepared Titanium reagents by a commercial laboratory (Research and Testing Laboratory, Lubbock, TX, USA). Sequences were deposited in NCBI SRA under BioProject PRJNA315365.

### Sequence Data Analyses

First, sequences were quality controlled by removing all reads that had a mismatch with the 16S rRNA primers, contained ambiguous nucleotides (N) and that were <270 nucleotides long after the forward primer. Then, a stringent quality trimming criteria was applied to remove reads having ≥3% of bases with Phred values <27 (0.2% per-base error probability). This is recommended to minimize erroneous reads when clustering at 97% (Huse et al., 2010; Kunin et al., 2010). Sequences were then de-replicated and clustered at a 97% threshold using Uclust (Edgar, 2010). Sequences from each OTU were classified by comparison to the Greengenes database (DeSantis et al., 2006). Sequence data analyses were conducted with Pyrotagger (Kunin and Hugenholtz, 2010). The taxonomic affiliations of the most abundant OTUs (>1% of the sequences) were further verified against sequences from the NCBI databases using BLAST (Altschul et al., 1990).

To compare bacterial communities for diversity analysis, all samples were randomly resampled to the size of the sample containing fewest sequences (*n* = 958) using Daisy Chopper (Gilbert et al., 2009). The Shannon diversity index (*H*′), the dominance index (*D*) and cluster analysis based on Bray–Curtis similarity were conducted using the software PAST (Hammer et al., 2001). Statistic comparisons of *H*′ and *D* between healthy and diseased sponge tissues were performed by Wilcoxon pair-test.

We also used PAST to conduct an analysis of similarities (ANOSIMs) using the Bray–Curtis distance to test if there were differences in community composition between groups of samples (Clarke et al., 2006).

A phylogenetic tree was constructed from the abundant OTUs and OTUs that showed significant changes in abundance between healthy and diseased tissues. Representative sequences for each OTU together with their best match from GenBank were aligned using MUSCLE (Edgar, 2004), and phylogenetic analyses were completed with the program PHYLIP (Felsenstein, 1989). DNADIST was used to calculate genetic distances using Kimura-2 model. The distance tree was estimated with the phylogenetic algorithm FITCH in the PHYLIP program. The significance of changes in phylum/class and OTU abundance was tested with a *t*-test and figures constructed with STAMP (Parks et al., 2014).

## Results

### Alpha and Beta Diversity

A total of 16 144 raw reads of the 16S rRNA gene were obtained from the sponge samples, and 16 642 row reads from the surrounding seawater. After removing poor-quality reads, a total of 12 842 reads remained (7532from sponge samples and 5310 from seawater). The total number of OTUs at a sequence similarity of 97% was 305 for the sponge tissues and 368 for the water.

The Shannon diversity index (*H*′) calculated from the resampled dataset was significantly higher (Wilcoxon pair-test, *p* < 0.05) in diseased samples compared to healthy samples (3.40 vs. 2.93 in average, respectively; **Table 1**). Conversely, the Simpson’s *D* was significantly higher (Wilcoxon pair test, *p* < 0.05) in healthy than in diseased tissues (0.10 and 0.07 in average, respectively; **Table 1**). Seawater samples had a higher number of OTUs than sponge tissues, whether healthy or diseased (**Table 1**), but significantly lower Shannon diversity than both types of sponge samples (Wilcoxon pair-test, *p* < 0.01 for both comparisons). Conversely the Simpson’s dominance index was significantly higher (Student *t*-test, *p* < 0.001) for seawater samples (*D* = 0.3 in average) than for sponges.

**Table 1:** Number of sequences, operational taxonomic units (OTUs), and diversity and dominance indices of bacterial communities from healthy and diseased sponge tissues, and surrounding seawater.

	All				Resampled			
		
	Sequences	OTUs	*H*′	*D*	Sequences	OTUs	*H*′	*D*
Healthy 1	1381	93	2.9	0.10	915	71	2.8	0.10
Healthy 2	1107	91	2.9	0.11	915	70	2.8	0.11
Healthy 3	1542	104	3.0	0.10	915	77	2.9	0.11
Diseased 1	1215	97	3.3	0.07	915	73	3.2	0.07
Diseased 2	1372	130	3.4	0.06	915	104	3.4	0.06
Diseased 3	915	119	3.4	0.07	915	119	3.4	0.07
Water 1	2433	223	2.5	0.32	915	107	2.4	0.31
Water 2	1849	196	2.6	0.31	915	113	2.39	0.32
Water 3	1028	148	2.6	0.27	915	104	2.4	0.31


*H′, Shannon index; D, Dominance.*

**FIGURE 2:**
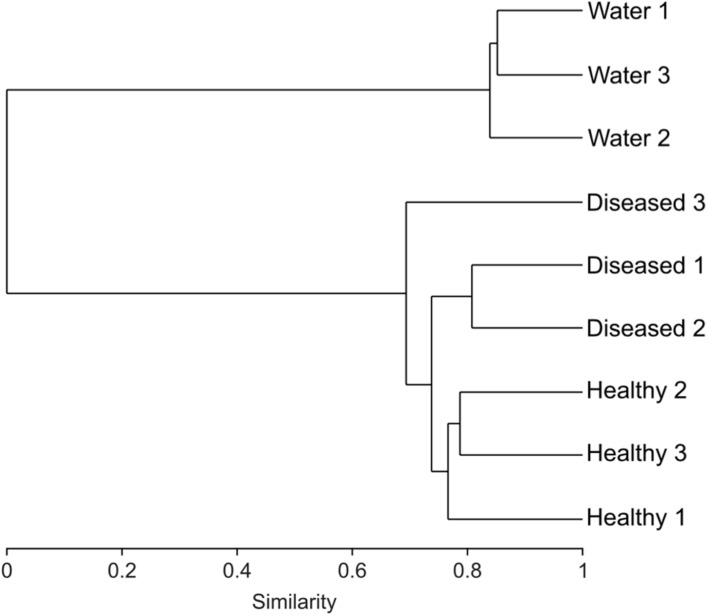
**Dendrogram based on the Bray–Curtis index showing the similarity between the 16S rRNA bacterial community for three diseased, healthy and surrounding seawater samples**.

**FIGURE 3:**
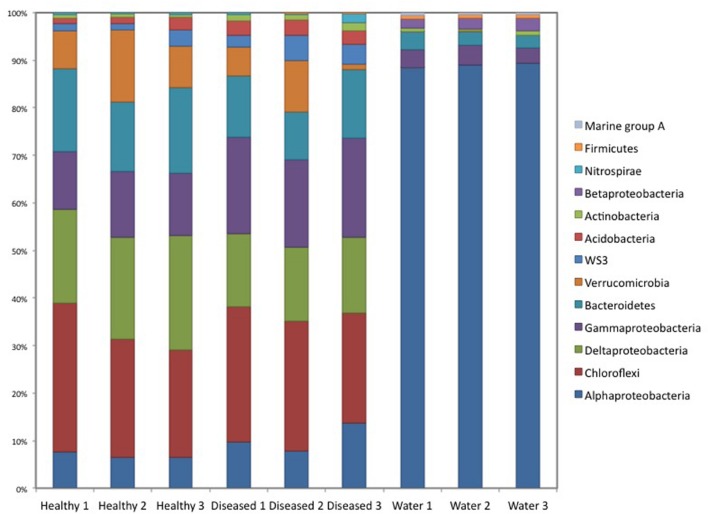
**Bacterial community composition for diseased and healthy sponge tissues, and the surrounding water**.

Cluster analysis of the samples (healthy and diseased sponges and water) based on their respective microbial community composition, using the Bray–Curtis similarity index, showed that the healthy tissues clustered together, apart from the diseased tissues (ANOSIM, *R* = 0.65; **Figure 2**),although one of the replicated samples of diseased tissue appeared as a sister group of both healthy and diseased. This replicate differed from the other two diseased samples by a lower number of *Verrucomicrobia* OTUs and the presence of Nitrospirae (**Figure 3**). The bacterial communities from the sponge tissues, either healthy or diseased, separated from that of seawater (ANOSIM, *R* = 1; **Figure 2**).

### Bacteria Community Composition in Sponges (Healthy and Diseased Tissues) vs. Seawater

**FIGURE 4:**
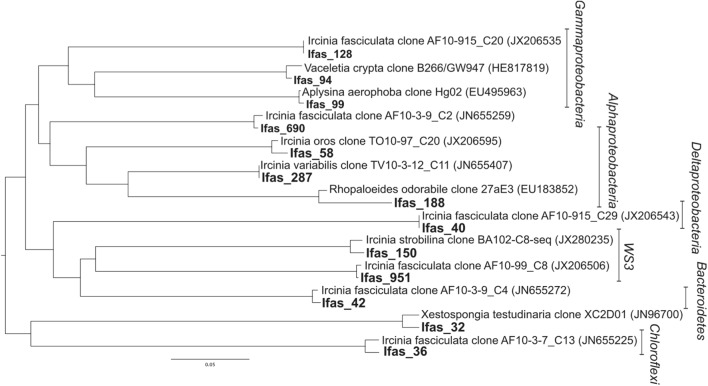
**Distance tree representing the position of the abundant operational taxonomic unit (OTUs; bold) found in *I. fasciculata* sponge tissues.** The best matching sequences from the database and their origin is specified.

Seawater samples were dominated by *Proteobacteria*, especially from the class *Alphaproteobacteria* (89% of the sequences; **Figure 3**), which belonged mainly to the SAR11 clade. *Bacteroidetes* was the second most abundant phylum in seawater samples. Sponge samples were also dominated by *Proteobacteria* but in this case mainly belonging to *Deltaproteobacteria* and *Gammaproteobacteria*. *Chloroflexi*, *Bacteroidetes*, and *Verrucomicrobia* were also abundant in sponge tissues (**Figure 3**). All abundant sponge sequences were similar to sequences known from the genus *Ircinia* or from other marine sponges belonging to several Porifera orders (e.g., *Aplysina aerophoba, Xestospongia testudinaria*, and *Haliclona hogarthi*). The most common *Deltaproteobacteria* (Ifas_40), *Chloroflexi* (Ifas_36)*, Bacteroidetes* (Ifas_42)and *Alphaproteobacteria* (Ifas_287) were identical to sequences from *Ircinia* species (Erwin et al., 2012a,b) (**Figure 4**), while the abundant *Verrucomicrobia* (Ifas_32) was similar to sequences from *X. testudinaria* (Montalvo and Hill, 2011).

### Healthy vs. Diseased Sponge Tissues

**FIGURE 5:**
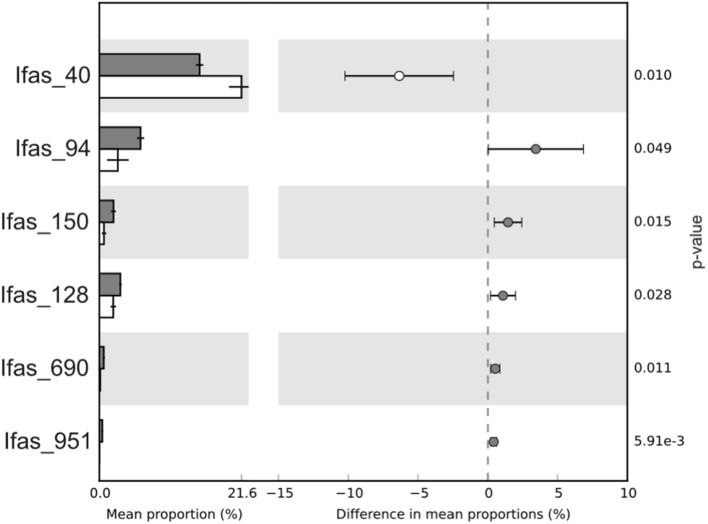
**Average proportion of OTUs that varied significantly in abundance between healthy (white bars) and diseased (gray bars) tissues.** Confidence intervals and *p*-values are indicated on the right side.

There were no qualitative differences between healthy and diseased tissues among the dominant groups at high taxonomical levels (phylum or class). However, a significant shift in abundance was detected for some groups. *Gammaproteobacteria*, *Acidobacteria*, and *Actinobacteria* increased in diseased tissues while *Deltaproteobacteria* were more abundant in healthy tissues (**Figure 3**). Changes were also detected at the OTU level (**Figure 5**). Among *Gammaproteobacteria* (**Figure 4**), OTUs Ifas_94 and Ifas_128 increased significantly in abundance in diseased tissues. Among *Deltaproteobacteria*, the OTU Ifas_40 (**Figure 4**) decreased in abundance in diseased tissues (**Figure 5**).

**FIGURE 6:**
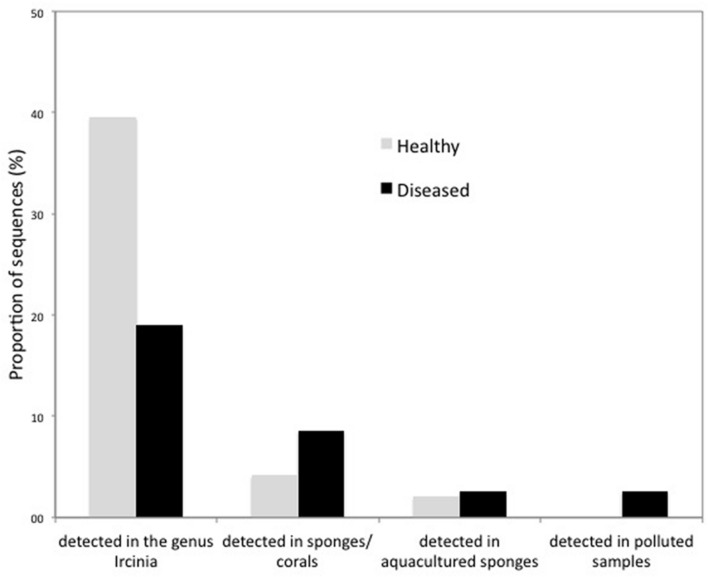
**Percentages of OTUs only recovered from healthy and diseased tissues of *I. fasciculata* that were previously described from the *Ircinia* genus, from diseased sponges or corals, from aquacultured sponges or from polluted samples**.

Qualitative differences between healthy and diseased sponges became relevant at lower taxonomical levels. More than 40% of the OTUs retrieved from healthy samples exclusively and 20% only found in the diseased samples had already been reported for the genus *Ircinia*. Furthermore, 8.6% of the bacteria only found in the diseased samples had already been associated to coral or sponge diseases, 2.6% to cultured sponges, and 2.6% to polluted environments (i.e., contaminated sediments or waste waters; **Figure 6**).

**FIGURE 7:**
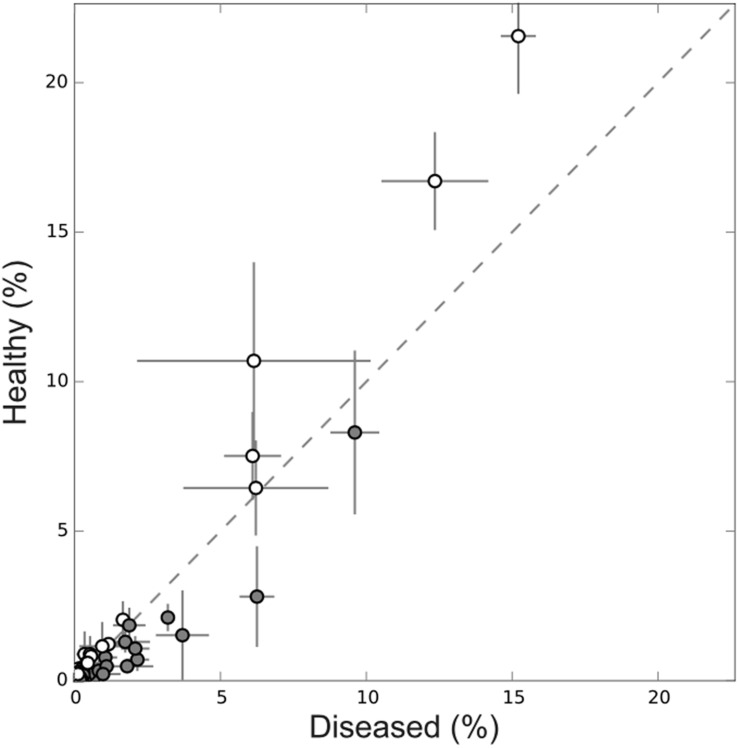
**Abundance plot of OTUs shared by healthy (empty symbol) and diseased (gray symbol) sponge tissues.** Abundant OTUs were more numerous in healthy sponges while in diseased tissues low abundance OTUs predominated.

Further, there was a higher proportion of abundant OTUs in healthy tissues while in diseased tissues less abundant OTUs predominated (**Figure 7**). More specifically, several OTUs significantly decreased in abundance in diseased samples. It was the case for the sponge most abundant OTU (Ifas_40) but also for other less abundant OTUs (**Figure 7**).

*Vibrio* sequences were rare. One *Vibrio* OTU (Ifas_224) was detected and was present only in the diseased samples (two sequences). It was100% similar to *Vibrio* sequences found earlier in both healthy and diseased sponge and coral species.

As for the phototrophic bacteria, a total of 23 Cyanobacteria OTUs were retrieved from both healthy and diseased *Ircinia* (remember that the *Candidatus Synechococcus spongiarum* layer was removed). The most abundant Cyanobacteria OTU (Ifas_228) was similar to sequences previously found in *Ircinia* (AF10-97_C30, Erwin et al., 2012b). This OTU, which was not detected in the water, was significantly more abundant in healthy than in diseased tissue.

## Discussion

Our study targeting healthy and early diseased individuals of *I. Fasciculata* revealed an alteration of the sponge bacterial community during the first phase of a Mediterranean massive sponge die-off caused by anomalous high temperatures (Cebrian et al., 2011). We hypothesize that the temperature-produced stress, to which the sponges were subjected, unbalanced the symbiotic bacterial community of healthy sponges causing changes in the sponge bacterial community composition and diversity. Seawater warming above normal values produces physiological stress in sponges (López-Legentil et al., 2008), which requires protein repair at the expenses of other vital functions (Agell et al., 2001). Stress has been reported to lower the sponge resistance against pathogens (Saby et al., 2009) and has been associated to infections (e.g., Klein, 1993). Thus, sponge stress likely caused the first bacterial community changes observed in early diseased sponges before opportunistic pathogens may operate. However, resistance to high temperatures and responses to consequent stress may be species–specific, as sublethal thermal stress did not produce a microbiome shift in the sponge *R. odorabile* until tissue necrosis was initiated (Simister et al., 2012).

One diseased sample differed from the other two with a lower number of *Verrucomicrobia* OTUs and the presence of *Nitrospira*, which could suggest that it represented a different stage of the disease, or simply variability in the samples. Conversely, an increase in *Verrucomicrobia* representatives has been recorded in diseased *Crella cyatophora* sponges from the Reed Sea (Gao et al., 2015), although, this enrichment might be due to the algae assemblages that covered the diseased sponge tissues (Gao et al., 2015).

At high taxonomical levels, the most abundant phyla in healthy *I. fasciculata* decreased in abundance in diseased tissues. Diseased samples showed less *Deltaproteobacteria* and more *Gammaproteobacteria* and *Acidobacteria* OTUs than healthy tissues. Shifts in sponge-associated bacteria community composition have been reported for other diseased sponges (reviewed in Webster, 2007; Gao et al., 2014, 2015), though changes seem to vary according to the sponge species and maybe the disease phase. Diseased individuals of *A. aerophoba* and *R. odorabile* showed higher abundances of *Bacteroidetes*, *Deltaproteobacteria*, and *Firmicutes*, and lower abundances of *Cyanobacteria*, *Chloroflexi*, and *Alpha*-and *Gammaproteobacteria* (Webster et al., 2008), a pattern shared by some corals (Cooney et al., 2002; Frias-Lopez et al., 2002).

Most of the OTUs exclusively retrieved from diseased tissues of *I. fasciculata* have been reported from other sponges, in particular of the genus *Ircinia*. Some OTUs were shared with cultured *Ircinia strobilina* and *Mycale laxissima* from the Caribbean (Mohamed et al., 2008a,b). Thus, phylogenetically close bacterial OTUs are present in healthy sponges from other geographical areas.

We observed higher bacterial diversity in diseased tissues. Increases in community diversity have also been recorded during sponge diseases in other oceans (Webster et al., 2008; Angermeier et al., 2011; but see Luter et al., 2010) and in cultured sponges (Mohamed et al., 2008a,b). Weakness of the sponge immunity system in stressed sponges, either because of culture conditions or anomalous high temperatures, combined with the appearance of new niches during protein degradation, may allow the presence of additional (not strictly symbiotic) bacteria, fostering bacterial diversity in unhealthy sponges.

Particular pathogens have been related to advanced stages of sponge diseases (Choudhury et al., 2015). A virus was reported to invade the diseased tissues of the sponge *Aplysina cavernicola* (Vacelet and Gallissian, 1978). Stabili et al. (2012) suggested that *V. rotiferianus* was involved in mortalities of *Ircinia* spp. from the South Adriatic and the Ionian Sea because it was isolated on agar plates from diseased sponge fragments, though its presence in the sponge tissues could not be confirmed. In our study, we detected only few sequences of one *Vibrio* OTU in both seawater and diseased tissue. Although, *Vibrio* spp. had been previously reported from other diseased sponges (Negandhi et al., 2010) and corals (e.g., Ben-Haim et al., 2003; Sussman et al., 2008), our results cannot demonstrate its direct implication in the mortality episodes of *I. fasciculata* as no significant increases in *Vibrio* sequences were detected in diseased tissues.

A decline in Cyanobacteria abundance was proposed to trigger the disease (Cebrian et al., 2011), which is likely since the absence of the Cyanobacteria colored layer was the most evident external symptom of the disease. However, it cannot be confirmed with our data as we removed on purpose the external Cyanobacteria-rich layer of the sponges to avoid our sequencing to be flooded by a single cyanobacterial OTU (see methods). Cyanobacteria are key components of the *I. fasciculata* microbiome (Erwin et al., 2012c) and may cover, as it has been reported for *Ircinia* sponges from other latitudes, a significant part of the species metabolic needs (Wilkinson, 1983). Thus, the sponge survival may be seriously compromised after Cyanobacteria decay in diseased tissues, leaving room for other components of the sponge microbiome to proliferate. Despites the absence of the external cyanobacterial layer, a total of 23 Cyanobacteria OTUs were retrieved from the inner part of the sponge and were significantly more abundant in healthy tissues. A Cyanobacteria shift was recorded earlier in the sponge *Xestospongia muta*, with healthy individuals mainly harboring *Synechococcus/Prochlorococcus* and orange-band-diseased individuals containing other Cyanobacteria previously associated with coral diseases, seawater, and sediments (Angermeier et al., 2011, 2012).

To summarize, as in previous studies of sponge diseases (Webster et al., 2008; Sweet et al., 2015), but in contrast to the unambiguous identification of a sponge pathogen in diseased *R. odorabile* from the Indo-Pacific (Choudhury et al., 2015), our study does not support the hypothesis of a sole pathogen, whether a *Vibrio* or any other bacteria, as the triggering factor of the Northwestern Mediterranean mass mortalities of *I. fasciculata*. Our results suggest a scenario where the bacterial community balance is disrupted in healthy sponges by a shift in the relative bacterial OTUs abundances and the consequent decrease of the sponge fitness, with later invasion of sponge-coral-specific opportunistic bacteria and purported pathogens.

## Author Contributions

All authors listed, have made substantial, direct and intellectual contribution to the work, and approved it for publication.

## Conflict of Interest Statement

The authors declare that the research was conducted in the absence of any commercial or financial relationships that could be construed as a potential conflict of interest.
